# Supplementary material for: Isolated pairs of Majorana zero modes in a disordered superconducting lead monolayer
Source: Nat Commun. 2019 Jun 13;10:2587. doi: 10.1038/s41467-019-10397-5 (PMC6565688; doi:10.1038/s41467-019-10397-5)
Supplement: Supplementary file 1 — Supplementary Information [file 41467_2019_10397_MOESM1_ESM.pdf]

Supplementary information for

"Isolated pairs of Majorana zero modes in a disordered superconducting lead monolayer"

Ménard et al.

Supplementary Notes

Supplementary Note 1: Fitting of the experimental zero mode peak

The shape of the central peak (Fig. 1g main text) can be fitted by a fermion-like mode convoluted by the Fermi-Dirac thermal broadening of $3.5k_\mathrm{B}T$ expected with a metallic STM tip. The width of the peak is well captured by an electronic temperature of 350 mK which is close to the 320 mK base temperature of our microscope (Suppl. Fig. S1a-b). Further we note that the conductance peak appears better fit with a slight shift from zero energy (Suppl. Fig. S1b). In general, due to the separation criterion one can not establish if the peak results from a single mode or a multiplet of modes in an energy range smaller than $3.5k_\mathrm{B}T$. The slight shift in energy position of the peak (Suppl. Fig. S1b) is certainly far smaller than $3.5k_\mathrm{B}T$, but we note that in general the energy position obtained with a fit to a single peak should correspond to the energy barycenter of an underlying multiplet of modes. Therefore, in accordance with theory, we attempt a fit by two modes (i.e., a sum of two peaks), and indeed find a better fit for a splitting energy $\pm 26\mu$eV with respective peak weights of 38% and 62% at negative and positive energy. The visual quality of these fits is demonstrated in Supplementary Figure 1c. Note that this fit is done using the base temperature of the microscope, not using an effective electronic temperature. Similar fitting attempts, with however a lower precision, can be done for spectra taken on the zero energy rim located at the edge, Fig. 1h in main text. One finds again an improvement through use of a split peak. Overall these fit attempts suggest that the data is consistent with a theoretical interpretation in terms of a very small energy splitting of the zero-bias modes due to a small overlap of their wave-functions in a finite size system. Note that from a general theoretical point of view, this splitting can be made arbitrarily small by increasing the spatial separation between the Majorana zero modes [1], and this indeed happens in our theoretical models when increasing the radius of island. We emphasize however that our experimental energy resolution is too small to consider as evidence any fitting beyond a single peak, so we offer this analysis as merely a guide for expected values of splitting energies to be confirmed in future experiments.

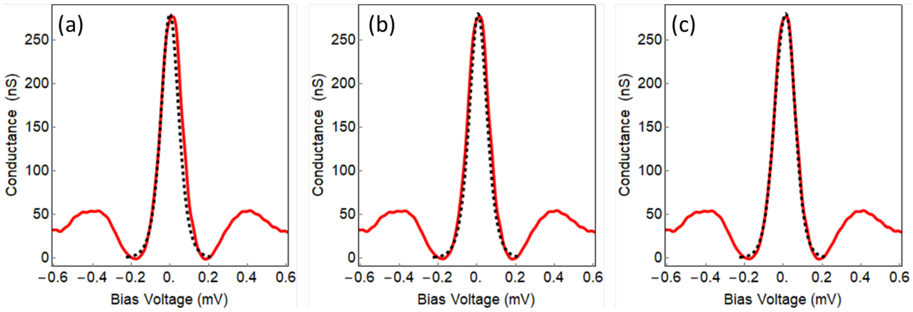

Supplementary Figure 1: Fitting of zero bias peak. (a,b), In (a) the zero bias peak of figure 1g in main text is fitted with a peak at zero energy convoluted by the derivative of the Fermi-Dirac distribution at 350 mK which is the estimated effective electronic temperature of the STM (base temperature 320 mK). The experimental peak appears shifted to positive energy. (b) Fit with same parameters as in (a) except that the peak energy is fixed to 6μeV. (c) Fit with a pair of peaks at $\pm 26\mu$eV with respective weights of 38% and 62% at negative and positive energy and an electronic temperature of 320 mK.

Supplementary Note 2: Other examples of observed pairs of zero modes

In Supplementary Figure 2 we show other examples of zero bias structures measured in Pb/Co/Si(111). As in Figure 1 of main text, the underlying Co-Si island domains do not appear in the topography but their effect is clearly visible in zero bias conductance maps. As in Figure 1 of main text, a strong zero bias peak is found in very small spot located in the middle of domains. A smaller zero bias peak is also found over an extended ring decaying very fast towards the inner domain and very slowly, over tens of nanometers, on the outside.

Supplementary Note 3: Ordinary superconducting vortex in Pb/Si(111)

The pair of zero bias peak structures we found at Co-Si domains cannot be explained by a zero energy mode of superconducting vortex core in a topological domain. Namely, in vortex cores generally one expects to have states with typical energy spacing of Δ^2/E_F. The total number of Caroli-Matricon-de Gennes bound states[2, 3] in a vortex core is of the order $N \approx E_\mathrm{F}/\Delta$, and here we have $E_\mathrm{F} \approx 660$ meV [4] and $\Delta \approx 0.35$ meV, expecting around 2000 bound states in a vortex core, i.e., a continuum of states. Moreover, our sample is in the ultradiffusive limit $\xi_\mathrm{eff} \gg l_e$, where

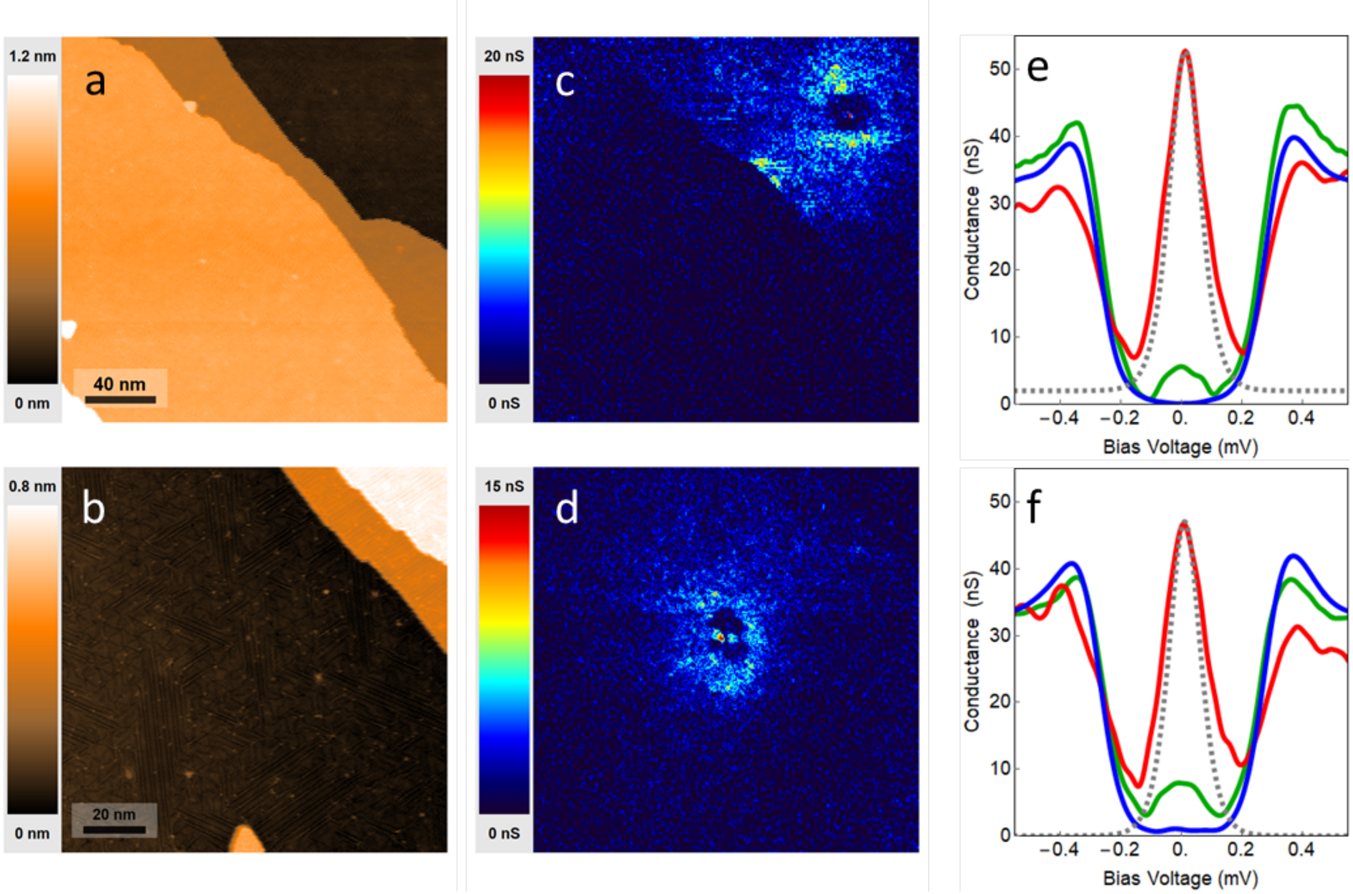

Supplementary Figure 2: Reproducible observations. (a,b), Scanning tunneling microscopy images of the sample showing a Pb monolayer with devil's staircase structure ($V_T = 50$ mV, $I_T = 50$ pA). The underlying Co-Si domain doesn't appear in the topography. **(c,d),** Corresponding conductance map at $V_T = 0$ mV shows an island-shaped domain with a strong zero bias peak (red dot) surrounded by a gapped region (dark blue) itself surrounded by a zero-bias rim (light blue). **(e,f),** Conductance curve taken at the center of the island domain shows a very high zero bias peak (red curve), fitted (dashed gray line) by a state at 10μV in **(e)** and 11μV in **(f)**. The spectra in green are taken on the light blue rim, they show a peak close to zero bias. The blue curves are reference conductance curves taken far from the island and show fully gapped BCS spectra.

$\xi_{\rm eff} \approx 50$ nm is the effective coherence length and $l_e \approx 2$ nm the mean free path. In this limit one expects to see superconducting vortices as normal state regions where the gap vanishes. This is indeed what we find in Pb/Si(111) monolayers [5], shown explicitly in Supplementary Figure 3. Note that the presence of a superconducting vortex has a big influence on the local density of states even very far from the vortex core. Further, a superconducting vortex is in general surrounded by strong screening currents due to the fact that the superconducting phase winds by 2π around the vortex. The radial superfluid velocity as function of the radius r is given by $v_s(r) = \frac{\hbar}{2m_e r}$, where m_e is the effective mass of the electrons. This superfluid velocity modifies the quasiparticle energy due to Doppler effect: $E_{\mathbf{k}} = \sqrt{\epsilon_k^2 + \Delta^2} + \hbar\mathbf{k}\cdot\mathbf{v}_s$ and this manifests by a broadening of the quasiparticle peaks at gap edge which is easily detected by STM[6]. In Supplementary Figure 3 we show that at a distance of 200 nm from the vortex core, which is equal to 4 times the coherence length, the local density of states does not recover the well developed hard gap that exists in the absence of a superconducting vortex. This is at sharp odds with what we found in Figure 1 of main text and Supplementary Figure 2, in which we don't find any gap closing nor the superfluid Doppler broadening (see the profile in Figure 1f of main text).

Supplementary Note 4: Modeling of the superconducting vortex

We consider a standard mean-field model of a superconducting vortex, having s-wave pairing $\Delta_{\rm S}(\mathbf{r}) \equiv \Delta_{\rm S}(1 - \exp(-|\mathbf{r}|/\xi))\exp(i\theta)$ suppressed at the origin where a single-winding vortex is introduced through polar angle θ. We

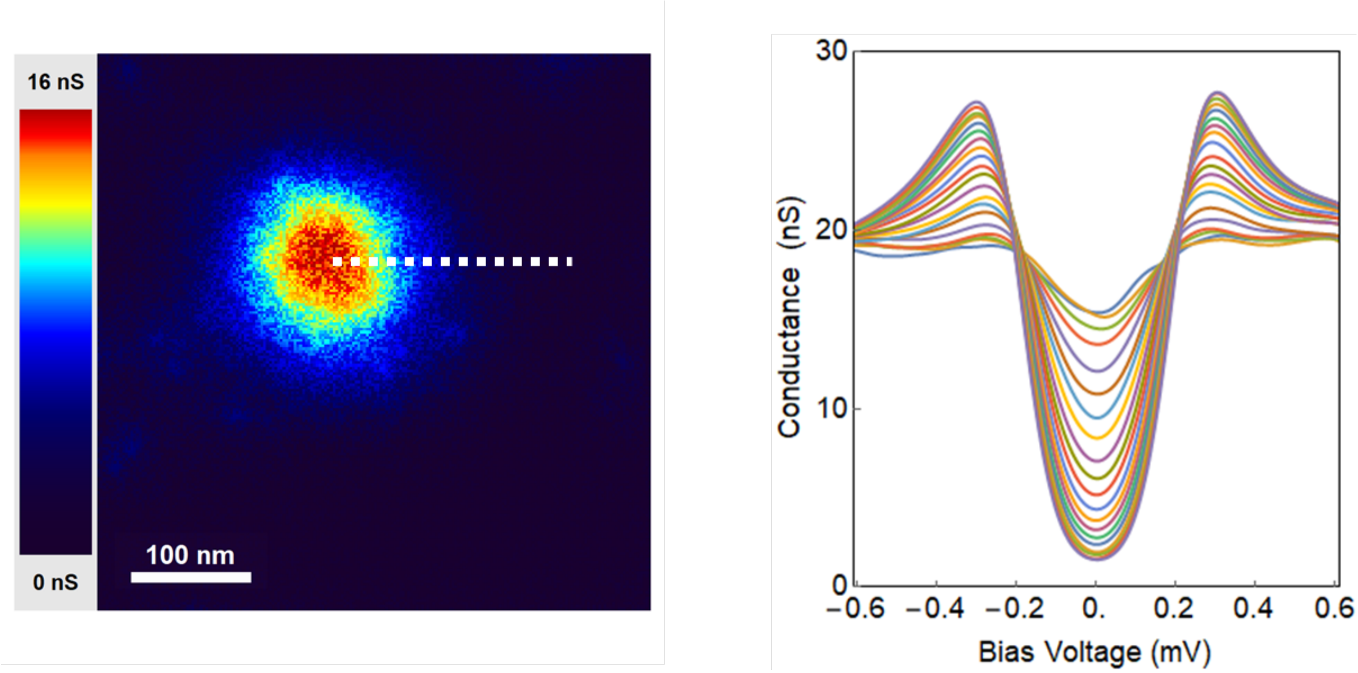

Supplementary Figure 3: Superconducting vortex observed. a, Scanning tunneling spectroscopy differential conductance map at $V_T = 0$ mV showing a superconducting vortex in a stripe incommensurate monolayer of Pb/Si(111) ($B = 40$ mT, $T = 320$mK). **b,** Conductance spectra taken along the dashed line in **a** showing the profile of the vortex. The spectra are taken every 10 nm from 0 to 200 nm away from the vortex center.

set the superconducting vortex into our general model of eq. 1 with a standard constant Rashba spin-orbit coupling of amplitude α:

$$\hat{H} = \int \mathrm{d}^2\mathbf{r}\ \Psi^\dagger_{\mathbf{r}} \left[(-\eta\nabla^2 - \mu - \mathrm{i}\alpha\sigma \times \nabla)\tau_z + V_z(\mathbf{r})\sigma_z + \Delta_{\mathrm{S}}(\mathbf{r})\tau_x\right] \Psi_{\mathbf{r}}. \tag{1}$$

We then treat this model numerically in the exact same way described in Methods and present the salient results in Supplementary Figure 4 which are directly comparable to the presented results for spin-orbit vortex because all the shared parameters in the two cases are set to the same values. Obviously, the superconducting vortex has many low-energy excitations extending on the largest lengthscale ξ, incompatible with the experimental observations in main text.

Supplementary Note 5: Theory of the spin-orbit vortex

There are two well-known spin-orbit terms induced in a plane (a material surface or heterostructure) due to breaking of inversion symmetry: the Rashba term and the Dresselhaus term. These terms still respect some point-group symmetries, namely, they share the symmetry of rotation by π around the z-axis, and a vertical mirror. The SIC system however breaks even these symmetries, allowing the additional spin-orbit coupling terms of the form $k_x\sigma_x$ or $k_y\sigma_y$, with (k_x, k_y) the electron in-plane momentum, and σ_α, $\alpha = x, y, z$ the electron spin operators. This leads to the possibility of the spin-orbit vortex. Consider first a simple mixing of the Rashba term with an $k_x\sigma_x + k_y\sigma_y$ term:

$$\mathcal{H}_{SOmix} = \alpha \left(\cos(\chi)\, \hat{z} \cdot (\sigma \times \mathbf{k}) - \sin(\chi)\, \sigma \cdot \mathbf{k}\right), \tag{2}$$

which in second quantization has the form:

$$\hat{H}_{\mathrm{SOmix}} = c^\dagger_{\mathbf{k}\uparrow}\, \alpha e^{\mathrm{i}\chi}(k_y + \mathrm{i}k_x)\, c_{\mathbf{k}\downarrow} + H.c., \tag{3}$$

where $c_{\mathbf{k}a}$ annihilates an electron of momentum $\mathbf{k} = (k_x, k_y)$ and spin z-component $a =\uparrow, \downarrow$.

In Supplementary eq. 3 it is obvious that the constant mixing angle χ plays the role of a phase of the Rashba spin-orbit coupling constant. We note that the constant χ does not influence the spectrum nor the pairing of electrons.

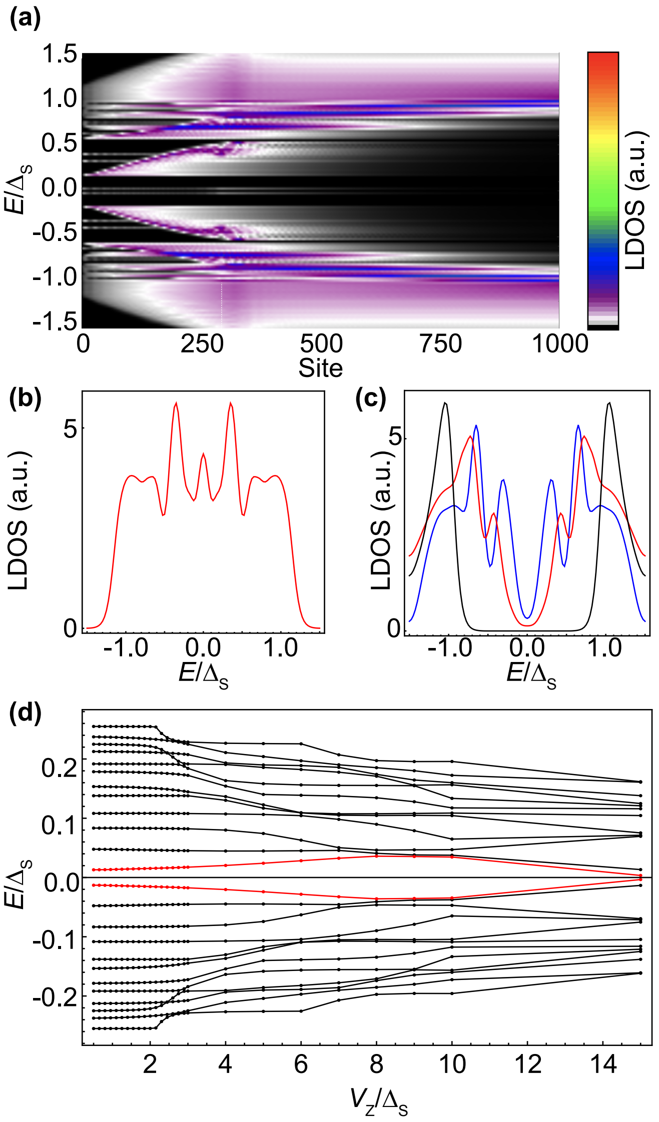

Supplementary Figure 4: Superconducting vortex and magnetic exchange. a, The local density of states(LDOS) of model Supplementary eq. 1 solved in circular geometry (see Methods and Supplementary Figure 5a) with single superconducting vortex at the center of a disk of radius $R = 300$ with constant magnetic exchange $V_z = 10\Delta_S$, and $V_z = 0$ outside. Model parameters match the ones used for spin-orbit vortex in Fig. 2: $(L, \xi/a, R/a, l_V/a, l_F/a, l_{SO}/a) = (8000; 400; 300; 80; 45; 3.3)$, and superconducting coherence length larger than island radius. **b,** Energy-dependent LDOS at origin of system in **a** (average of sites in radius 3% of magnetic disk radius). Thermal broadening is simulated by convolution with derivative of Fermi-Dirac distribution at temperature $k_B T/\Delta_S = 0.1$. **c,** Energy-dependent LDOS at edge R of magnetic disk (red line) in **a** (average within edge ring of width 3% of magnetic disk radius), in a similar ring within disk at distance $R/2$ (blue), and reference LDOS far outside magnetic disk (black). **d,** Spectrum of excitations in superconductor of pairing energy Δ_S for superconducting vortex—anti-vortex pair in a plane of size $L = 450$ with periodic boundary conditions and with constant magnetic exchange V_z, and all common model parameters match Fig. 1e, i.e., $(L, \xi/a, l_V/a, l_F/a, l_{SO}/a) = (450; 80; 8; 1.9; 0.7)$ (see Methods and Supplementary Figure 5). The lowest 22 energies are plotted for each V_z, with lowest two in red.

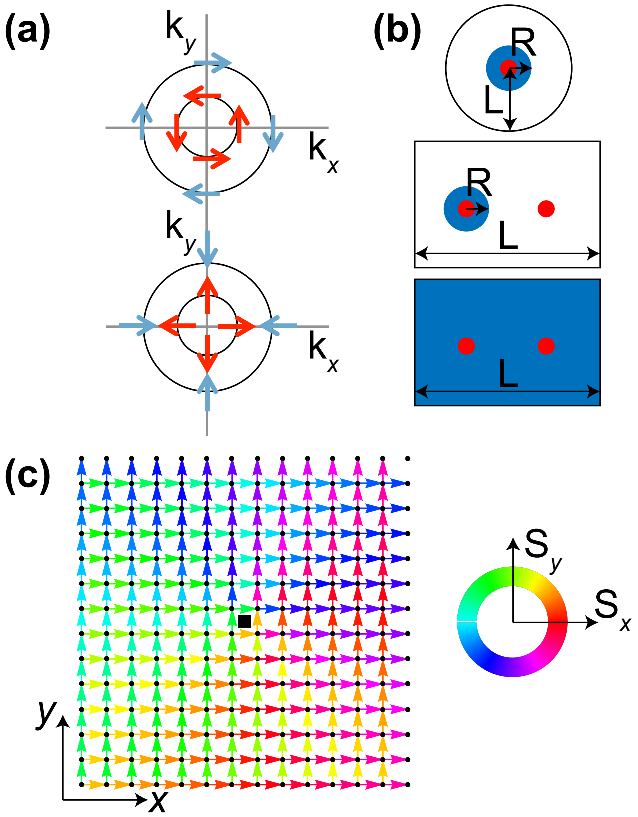

Supplementary Figure 5: Spin-orbit vortex definition and system geometries. a, Top: Two Fermi surfaces (black circles) of model in eq. 1 without any vortex but with constant Rashba spin-orbit coupling. Arrows mark the spin direction at several momenta. The momentum-spin locking is compatible with singlet pairing of opposite points on either Fermi surface. Bottom: The same when Rashba spin-orbit coupling is replaced by $\mathbf{k}\cdot\sigma$. The same singlet pairing is still compatible. **b,** Schematic system geometries used in numerical calculations of vortices (spin-orbit or superconducting). Red dots are vortex positions. Magnetic exchange is non-zero in blue areas, and zero in white. Top: circular geometry with open boundary conditions. Middle, Bottom: torus geometry, periodic boundary conditions on rectangle. **c,** Square lattice tight-binding representation of 2π-winding spin-orbit vortex (located at black rectangle). Arrow marks direction of hopping between nearest neighbors, while color of arrow describes spin Pauli matrix that is applied to that hopping (color code on circle), thereby determining the local momentum-spin locking. Note the center-right area of lattice (purple-red) with Rashba spin-orbit pattern $k_y\sigma_x - k_x\sigma_y$, and the bottom-center area (red-green) with pattern $k_x\sigma_x + k_y\sigma_y$.

The simple 2π spin-orbit vortex is obtained by making the mixing *local*, i.e., $\chi \to \chi(x,y)$, and allowing the phase $\chi(x,y)$ to wind by 2π around a singular point (see Supplementary Figure 5.c), i.e.,

$$\chi(x,y) = \theta(x,y), \tag{4}$$

where θ is the polar angle in the plane.

Explicitly, the Hamiltonian of the 2π spin-orbit vortex, written in the σ_z basis of Supplementary eq. 2 using polar coordinates, becomes:

$$\mathcal{H}_{\text{SO-vortex}} = \alpha \begin{pmatrix} 0 & \partial_r - \frac{\mathrm{i}}{r}\partial_\theta + \frac{1}{2r} \\ -\partial_r - \frac{\mathrm{i}}{r}\partial_\theta - \frac{1}{2r} & 0 \end{pmatrix}. \tag{5}$$

The spin-orbit vortex obviously shifts the angular momentum to integer values, which allows protected zero-energy states at angular momentum zero.

We note that the s-wave singlet pairing of $|\mathbf{k}\uparrow\rangle$ and $|-\mathbf{k}\downarrow\rangle$ is compatible with either of the two spin-orbit terms (Supplementary eq.2) that we locally mix in the vortex (see Supplementary Figure 5.a). Therefore the spin-orbit vortex is a defect fully contained in the kinetic energy (not in pairing), and also it is not expected to inhibit pairing.

The spin-orbit vortex model of Eq. 1 with a constant $V_z(x,y) = V_z$ in the entire plane has been considered exclusively focusing on the Majorana state at zero energy and zero angular momentum[7, 8]. In this subspace, finding the rescaled Majorana wavefunction $\tilde{\psi} = \psi/\sqrt{r}$, with r the radial coordinate, reduces to solving

$$\begin{pmatrix} -\partial_s^2 - \frac{1}{4s^2} + \tilde{V} & \partial_s + \lambda\tilde{\Delta}_{\mathrm{S}} \\ -\partial_s - \lambda\tilde{\Delta}_{\mathrm{S}} & -\partial_s^2 - \frac{1}{4s^2} - \tilde{V} \end{pmatrix} \tilde{\psi}(s) = 0, \tag{6}$$

with dimensionless parameters $s = r/\alpha$, $\tilde{\Delta}_{\mathrm{S}} = \Delta_{\mathrm{S}}/\alpha^2$, $\tilde{V}_z = V_z/\alpha^2$, $\mu = 0$, and $\lambda = \pm 1$. The presence of multiple physical lengthscales, i.e., $\xi \sim 1/\Delta_{\mathrm{S}}$, $l_V \sim 1/V_Z$ and $l_{\mathrm{SO}} \sim 1/\alpha$, makes even this simple model analytically inaccessible[7, 8].

In this work we instead focus on numerically studying both the zero-energy state and the excitation energies using several geometries and versions of the model, including the case of V_z being non-zero only within a disk representing the Co island.

Supplementary Note 6: Magnetic exchange texture model

To present the similarities between the spin-orbit vortex and a magnetic exchange texture in two dimensions, we start from the explicit matrix form of the continuum radial model of the spin-orbit vortex, i.e., eq. 1 right before discretization in eq. 4:

$$H_{\mathrm{SOvortex}} = H_0 + \delta H_{\mathrm{SOvortex}}, \tag{7}$$

with

$$H_0 = -\eta\left(\partial_r^2 + \frac{1-4m^2}{4r^2}\right)\tau_z + \Delta_{\mathrm{S}}\tau_x + V_z\sigma_z, \tag{8}$$

and

$$\begin{aligned} \delta H_{\mathrm{SOvortex}} =& \alpha(i\partial_r)\sigma_y\tau_z + \\ &+ \alpha\frac{m}{r}\sigma_x\tau_z, \end{aligned} \tag{9}$$

where m is an integer angular momentum, and we assume a disk-shaped system of radius L with open boundary. The magnetic exchange V_z is constant throughout the system. Next we consider a magnetic texture continuum model of eqs. 1 and 3, removing any spin-orbit coupling term for simplicity. General magnetic exchange (or Zeeman field) textures $\mathbf{V}$ will be considered elsewhere, but here we postulate a skyrmion, $\mathbf{V} = V_0[\cos(f_r)\cos(n\theta), \cos(f_r)\cos(n\theta), \sin(f_r)]$, with θ the polar angle and n an integer, and we simplify by taking $2f_r \equiv A + \beta r$. To reduce to radial form, first note that if n is even the total angular momentum is integer valued, $m + \frac{n}{2}$, with m an integer. We reduce Hamiltonian to radial form using this, and then apply a local rotation of electron until the remaining explicit texture term $\mathbf{V}_{\mathrm{rad}} \cdot \sigma$ becomes constant in space, $V_0\mathbf{e_z}$. The explicit transformation for reduction to radial form is $\Psi_{r,\theta} \equiv \frac{1}{\sqrt{r}}\exp\left(\mathrm{i}(m\theta - \frac{n}{2}\sigma_z)\right)\exp\left(-\mathrm{i}f_r\sigma_y\right)$, giving:

$$H_{\mathrm{texture}} = H_0 + \delta H_{\mathrm{texture}}, \tag{10}$$

with

$$\begin{aligned} \delta H_{\mathrm{texture}} =& \eta\beta\left[(\mathrm{i}\partial_r)\sigma_y + \frac{\beta}{4}\right]\tau_z + \\ &+ \eta\beta\frac{m}{r}\left[K\sigma_x + \frac{n}{r}\left(\sin(2f_r)\sigma_x - \cos(2f_r)\sigma_z\right)\right]\tau_z, \end{aligned} \tag{11}$$

where the single correction term proportional to K (in the second line) was added by hand for later discussion.

One sees a resemblance between the H_{SOvortex} and H_{texture}, with radial winding of the skyrmion, measured by β, inducing an effective spin-orbit coupling. Ref.[9] pointed out that H_{texture} (without correction, $K = 0$) has a central Majorana zero mode for β large enough, but also many other low energy excitations, in contrast to H_{SOvortex}.

We find that all the unwanted excitations of the skyrmion model can be removed from the low-energy spectrum (below Δ_{S}) by the addition of single correction term, $K \neq 0$, see Supplementary Figure 7. The correction is simply

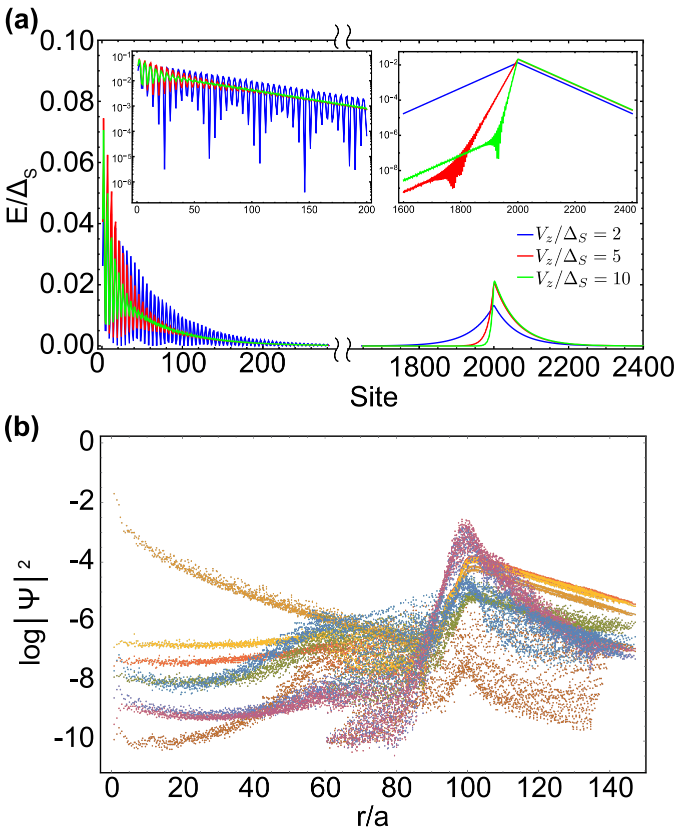

Supplementary Figure 6: Spin-orbit vortex on island: magnetic exchange dependence and excitations. a, The radial dependence of local density of states(LDOS) at zero energy in the spin-orbit vortex model of eq. 1 solved in circular geometry (see Methods for definitions and technical details) with single spin-orbit vortex at the center of a disk (radius $R = 2000$) with constant magnetic exchange V_z (see legend), and $V_z = 0$ outside. Model parameters are $(L, \xi/a, R/a, l_{\rm F}/a, l_{\rm SO}/a) = (10000; 80; 2000; 1.9; 0.7)$. Here, the superconducting coherence length is smaller than the island radius. Inset left: Zoom-in on center of disk, on logarithmic scale, showing the strong localization at spin-orbit vortex, but also a slowly decaying tail. Inset right: Zoom-in to disk edge region, on logarithmic scale. Note the asymmetric decay on inside and outside. This LDOS is contributed by exactly two Majorana zero-mode wavefunctions. **b,** The radial decay, from the position of spin-orbit vortex, of angularly averaged wavefunction amplitudes of the lowest 16 excited states in a two-dimensional tight-binding version of model eq. 1 with periodic boundary conditions, a non-zero magnetic exchange $V_z/\Delta_{\rm S} = 10$ on a disk of radius $R/a = 100$ centered at the spin-orbit vortex at origin, while other parameters are $(L, \xi/a, R/a, l_{\rm F}/a, l_{\rm SO}/a) = (300; 20; 100; 1.9; 0.7)$ (see Methods for definitions and technical details). The two zero energy wavefunctions appear identical (the only curve peaking at origin), while all excited states are island edge states. Here, the superconducting coherence length is smaller than island radius R.

the term occurring in $H_{\rm SOvortex}$, second line of Supplemenatry eq. 9. Upon adding the correction term ($K \neq 0$), the induced chemical potential, the choice of (any even) n, and exact shape of f_r in $H_{\rm texture}$ do not play a crucial role for the spectrum.

Importantly, the correction term $K \cdot \left(\frac{m}{r}\sigma_x\tau_z\right)$ in $H_{\rm texture}$ is of the exact same matrix form as a term already induced by the skyrmion, namely, $\frac{n\sin(2f_r)}{r} \cdot \left(\frac{m}{r}\sigma_x\tau_z\right)$. The correction term forbids the non-zero-mode low-energy excitations because it does not have the spatially decaying factor of the skyrmion term, $\frac{\sin(2f_r)}{r}$. Interestingly, this factor does not decay spatially in the limit of small f_r, i.e., small β and $A = 0$, but this limit is contrary to the necessity of large β to increase the induced spin-orbit coupling. This indicates that skyrmion-like textures should be considered beyond the continuum limit; in principle, it seems there should be various textures which could compensate the undesirable decay with r from the outset, and we leave this kind of study for future work.

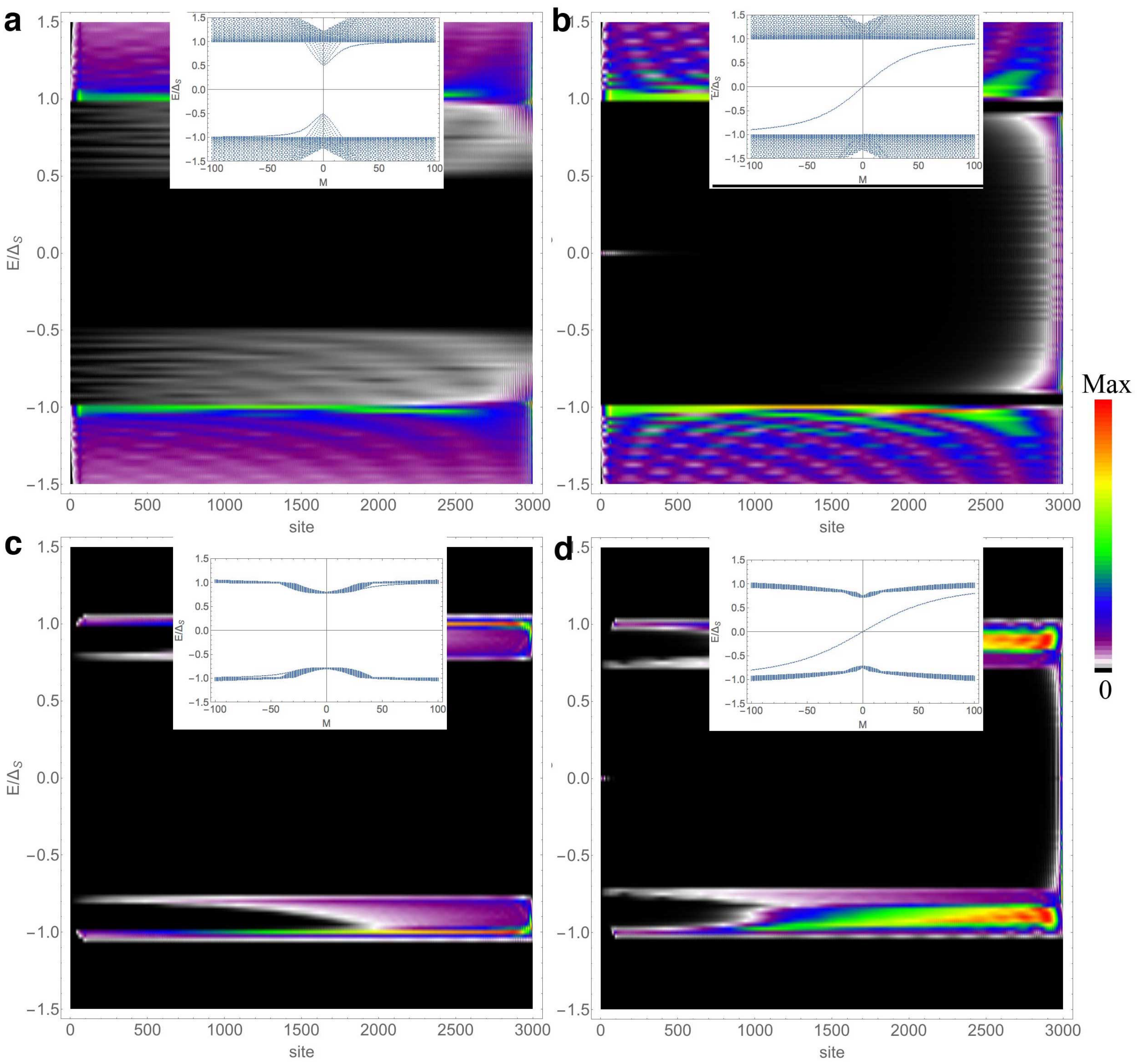

Supplementary Figure 7: Magnetic exchange texture vs. spin-orbit vortex. a, The local density of states(LDOS) of model 7, i.e., the model of eq. 1 solved in circular geometry with single spin-orbit vortex at the origin and a constant magnetic exchange $V_z = 0.5\Delta_S$ in the non-topological regime. Model parameters are $(L, \xi/a, l_F/a, l_{SO}/a) = (3000; 80; 1.9; 0.7)$. Apart from bulk states due to the nearby topological transition at $V_z = \Delta_S$, there are no excitations below Δ_S. Inset: The spectrum as function of angular momentum m, with 40 lowest states at each m. **b,** and inset: The same spin-orbit vortex system as in **a**, but in topological regime, $V_z = 2\Delta_S$. There are two Majorana wavefunctions at $m = 0$, one localized at origin and one at edge. There is a clear chiral branch of edge modes at the edge of this topological system. The edge modes are dense in the gap due to their very large radius (the system radius L). **c,** The local density of states(LDOS) of corrected skyrmion texture model 10 with $K = 1$, $n = 2$ solved in circular geometry in the non-topological regime $V_0 = 0.5\Delta_S$. Model parameters are $(L, \xi/a, A, \beta) = (3000; 80; \pi/2; 1000)$. There are no excitations below $\sim \Delta_S$. Inset: The spectrum as function of angular momentum m, with 40 lowest states at each m. **d,** and inset: The same texture system as in **c**, but in topological regime, $V_0 = 2\Delta_S$. The spectral features are the same as in the spin-orbit vortex model of **b**.

Supplementary References

[1] Alicea, J. New directions in the pursuit of Majorana fermions in solid state systems. *Reports on Progress in Physics* **75**, 6501 (2012).

[2] Caroli, C., De Gennes, P. G. & Matricon, J. Bound Fermion states on a vortex line in a type II superconductor. *Physics Letters* **9**, 307–309 (1964).

[3] Kopnin, N. B. & Salomaa, M. M. Mutual friction in superfluid 3He: Effects of bound states in the vortex core. *Physical Review B (Condensed Matter)* **44**, 9667–9677 (1991).

[4] Brand, C. *et al.* Spin-resolved band structure of a densely packed Pb monolayer on Si(111). *Physical Review B* **96**, 035432 (2017).

[5] Brun, C. *et al.* Remarkable effects of disorder on superconductivity of single atomic layers of lead on silicon. *Nature Physics* **10**, 444–450 (2014).

[6] Kohen, A. *et al.* Probing the superfluid velocity with a superconducting tip: The Doppler shift effect. *Physical Review Letters* **97**, 027001 (2006).

[7] Sato, M., Takahashi, Y. & Fujimoto, S. Non-Abelian topological order in s-wave superfluids of ultracold fermionic atoms. *Physical Review Letters* **103**, 020401 (2009).

[8] Sau, J. D., Tewari, S., Lutchyn, R. M., Stanescu, T. D. & Das Sarma, S. Non-Abelian quantum order in spin-orbit-coupled semiconductors: Search for topological Majorana particles in solid-state systems. *Physical Review B* **82**, 214509 (2010).

[9] Yang, G., Stano, P., Klinovaja, J. & Loss, D. Majorana bound states in magnetic skyrmions. *Physical Review B* **93**, 224505 (2016).
